# Supplementary material for: Novel potential of low calorie plant burger: Functional turkey meat formulation optimized by replacing quinoa, chia, soy, amaranth and peas as vegetable protein and their influence on texture and sensory traits
Source: PLoS One. 2025 Jul 23;20(7):e0325622. doi: 10.1371/journal.pone.0325622 (PMC12286408; doi:10.1371/journal.pone.0325622)
Supplement: S1 File — (ZIP) [file pone.0325622.s001.zip › CCD.rtf]

WORKSHEET 1
Response Optimization: Total acceptability, b*, a*, L*, Adhesiveness, Chewiness, Guminess, Elasticity, Cohesiveness, Hardness
Parameters
Response	Goal	Lower	Target	Upper	Weight	Importance	
Total acceptability	Maximum	3.40	4.950	 	1	1	
b*	Minimum	 	4.230	5.980	1	1	
a*	Minimum	 	1.450	2.870	1	1	
L*	Maximum	81.23	95.540	 	1	1	
Adhesiveness	Minimum	 	-7.820	-4.320	1	1	
Chewiness	Minimum	 	1.540	3.220	1	1	
Guminess	Minimum	 	2.350	3.360	1	1	
Elasticity	Minimum	 	0.521	0.623	1	1	
Cohesiveness	Minimum	 	0.255	0.351	1	1	
Hardness	Minimum	 	9.870	19.720	1	1	
Solution
Solution	Quinoa	Soybean	Amaranth	Total
acceptability
Fit	b*
Fit	a*
Fit	L*
Fit	Adhesiveness
Fit	
1	25	11.8687	25	4.41124	4.38585	1.51237	92.9280	-7.62024	
Solution	Chewiness
Fit	Guminess
Fit	Elasticity
Fit	Cohesiveness
Fit	Hardness
Fit	Composite
Desirability	
1	1.64949	2.35034	0.594016	0.261141	9.70303	0.801103	
Multiple Response Prediction
Variable	Setting	
Quinoa	25	
Soybean	11.8687	
Amaranth	25	
Response	Fit	SE Fit	95% CI	95% PI	
Total acceptability	4.411	0.237	(3.883, 4.940)	(3.593, 5.229)	
b*	4.386	0.166	(4.016, 4.756)	(3.813, 4.959)	
a*	1.5124	0.0607	(1.3771, 1.6477)	(1.3028, 1.7219)	
L*	92.928	0.687	(91.397, 94.459)	(90.558, 95.298)	
Adhesiveness	-7.620	0.170	(-7.999, -7.241)	(-8.207, -7.033)	
Chewiness	1.649	0.115	(1.393, 1.906)	(1.252, 2.047)	
Guminess	2.3503	0.0400	(2.2612, 2.4395)	(2.2123, 2.4884)	
Elasticity	0.59402	0.00833	(0.57545, 0.61259)	(0.56526, 0.62277)	
Cohesiveness	0.26114	0.00613	(0.24747, 0.27481)	(0.23998, 0.28231)	
Hardness	9.703	0.326	(8.976, 10.430)	(8.578, 10.828)	

Multiple Response Prediction
Variable	Setting	
Quinoa	25	
Soybean	11.8687	
Amaranth	0	
Response	Fit	SE Fit	95% CI	95% PI	
Total acceptability	4.550	0.237	(4.022, 5.079)	(3.732, 5.368)	
b*	4.805	0.166	(4.435, 5.174)	(4.232, 5.377)	
a*	1.9386	0.0607	(1.8032, 2.0739)	(1.7290, 2.1481)	
L*	86.499	0.687	(84.968, 88.029)	(84.128, 88.869)	
Adhesiveness	-6.612	0.170	(-6.991, -6.233)	(-7.198, -6.025)	
Chewiness	2.256	0.115	(2.000, 2.513)	(1.859, 2.654)	
Guminess	2.4795	0.0400	(2.3904, 2.5687)	(2.3415, 2.6176)	
Elasticity	0.55181	0.00833	(0.53324, 0.57038)	(0.52305, 0.58057)	
Cohesiveness	0.28710	0.00613	(0.27344, 0.30077)	(0.26594, 0.30827)	
Hardness	11.973	0.326	(11.247, 12.700)	(10.848, 13.099)	
