# Supplementary material for: Novel potential of low calorie plant burger: Functional turkey meat formulation optimized by replacing quinoa, chia, soy, amaranth and peas as vegetable protein and their influence on texture and sensory traits
Source: PLoS One. 2025 Jul 23;20(7):e0325622. doi: 10.1371/journal.pone.0325622 (PMC12286408; doi:10.1371/journal.pone.0325622)
Supplement: S1 File — (ZIP) [file pone.0325622.s001.zip › Taguchi/Emulsion stability.rtf]

WORKSHEET 1
Taguchi Analysis: Emulsion stability versus A, B, C, D, E
Response Table for Signal to Noise Ratios
Nominal is best (10×Log10(Ybar^2/s^2))
Level	A	B	C	D	E	
1	*	*	*	*	*	
2	*	*	*	*	*	
Delta	*	*	*	*	*	
Rank	3	3	3	3	3	
Response Table for Means
Level	A	B	C	D	E	
1	28.89	30.52	29.55	31.49	27.56	
2	37.75	36.12	37.09	35.15	39.07	
Delta	8.85	5.59	7.54	3.66	11.51	
Rank	2	4	3	5	1	

* ERROR * No graphs will be plotted for SN ratios. All values are missing.
