# Supplementary material for: Novel potential of low calorie plant burger: Functional turkey meat formulation optimized by replacing quinoa, chia, soy, amaranth and peas as vegetable protein and their influence on texture and sensory traits
Source: PLoS One. 2025 Jul 23;20(7):e0325622. doi: 10.1371/journal.pone.0325622 (PMC12286408; doi:10.1371/journal.pone.0325622)
Supplement: S1 File — (ZIP) [file pone.0325622.s001.zip › Taguchi/Fiber.rtf]

WORKSHEET 1
Taguchi Analysis: Fiber (%) versus A, B, C, D, E
Response Table for Signal to Noise Ratios
Nominal is best (10×Log10(Ybar^2/s^2))
Level	A	B	C	D	E	
1	*	*	*	*	*	
2	*	*	*	*	*	
Delta	*	*	*	*	*	
Rank	3	3	3	3	3	
Response Table for Means
Level	A	B	C	D	E	
1	0.6900	0.5850	0.5750	0.5750	0.5750	
2	0.2500	0.3550	0.3650	0.3650	0.3650	
Delta	0.4400	0.2300	0.2100	0.2100	0.2100	
Rank	1	2	3.5	5	3.5	

* ERROR * No graphs will be plotted for SN ratios. All values are missing.
