# Supplementary material for: Novel potential of low calorie plant burger: Functional turkey meat formulation optimized by replacing quinoa, chia, soy, amaranth and peas as vegetable protein and their influence on texture and sensory traits
Source: PLoS One. 2025 Jul 23;20(7):e0325622. doi: 10.1371/journal.pone.0325622 (PMC12286408; doi:10.1371/journal.pone.0325622)
Supplement: S1 File — (ZIP) [file pone.0325622.s001.zip › Taguchi/Moisture.rtf]

WORKSHEET 1
Taguchi Analysis: Moisture (%) versus A, B, C, D, E
Response Table for Signal to Noise Ratios
Nominal is best (10×Log10(Ybar^2/s^2))
Level	A	B	C	D	E	
1	*	*	*	*	*	
2	*	*	*	*	*	
Delta	*	*	*	*	*	
Rank	3	3	3	3	3	
Response Table for Means
Level	A	B	C	D	E	
1	33.40	34.23	34.27	34.41	34.45	
2	22.27	21.43	21.39	21.25	21.21	
Delta	11.13	12.81	12.89	13.17	13.23	
Rank	5	4	3	2	1	

* ERROR * No graphs will be plotted for SN ratios. All values are missing.
