# Supplementary material for: Novel potential of low calorie plant burger: Functional turkey meat formulation optimized by replacing quinoa, chia, soy, amaranth and peas as vegetable protein and their influence on texture and sensory traits
Source: PLoS One. 2025 Jul 23;20(7):e0325622. doi: 10.1371/journal.pone.0325622 (PMC12286408; doi:10.1371/journal.pone.0325622)
Supplement: S1 File — (ZIP) [file pone.0325622.s001.zip › Taguchi/OHC.rtf]

WORKSHEET 1
Taguchi Analysis: OHC (g/g) versus A, B, C, D, E
Response Table for Signal to Noise Ratios
Nominal is best (10×Log10(Ybar^2/s^2))
Level	A	B	C	D	E	
1	*	*	*	*	*	
2	*	*	*	*	*	
Delta	*	*	*	*	*	
Rank	3	3	3	3	3	
Response Table for Means
Level	A	B	C	D	E	
1	2.560	2.805	2.560	2.705	2.300	
2	3.610	3.365	3.610	3.465	3.870	
Delta	1.050	0.560	1.050	0.760	1.570	
Rank	3	5	2	4	1	

* ERROR * No graphs will be plotted for SN ratios. All values are missing.
