# Supplementary material for: Novel potential of low calorie plant burger: Functional turkey meat formulation optimized by replacing quinoa, chia, soy, amaranth and peas as vegetable protein and their influence on texture and sensory traits
Source: PLoS One. 2025 Jul 23;20(7):e0325622. doi: 10.1371/journal.pone.0325622 (PMC12286408; doi:10.1371/journal.pone.0325622)
Supplement: S1 File — (ZIP) [file pone.0325622.s001.zip › Taguchi/protein q.rtf]

WORKSHEET 1
Taguchi Analysis: Protein (%) versus A, B, C, D, E
Response Table for Signal to Noise Ratios
Nominal is best (10×Log10(Ybar^2/s^2))
Level	A	B	C	D	E	
1	*	*	*	*	*	
2	*	*	*	*	*	
Delta	*	*	*	*	*	
Rank	3	3	3	3	3	
Response Table for Means
Level	A	B	C	D	E	
1	53.18	50.89	51.09	50.90	50.88	
2	64.39	66.68	66.48	66.67	66.69	
Delta	11.22	15.79	15.40	15.78	15.81	
Rank	5	2	4	3	1	

* ERROR * No graphs will be plotted for SN ratios. All values are missing.
